# Different Dynamics: *Meta*-Topolin Induces Faster and More Pronounced Upregulation of Selected Shoot Development-Related Genes in *Arabidopsis thaliana* Compared to *Trans*-Zeatin and Thidiazuron

**DOI:** 10.3390/biology15171499

**Published:** 2026-09-02

**Authors:** Nina Pokimica, Tatjana Ćosić, Slavica Ninković, Aleksandar Cingel, Václav Motyka, Milorad Vujičić, Martin Raspor

**Affiliations:** 1 Department of Plant Physiology, Institute for Biological Research “Siniša Stanković”—National Institute of Republic of Serbia, University of Belgrade, Bulevar Despota Stefana 142, 11108 Belgrade, Serbia; 2 Laboratory of Hormonal Regulations in Plants, Institute of Experimental Botany of the Czech Academy of Sciences, Rozvojová 263, 16502 Prague, Czech Republic; 3 Institute of Botany and Botanical Garden “Jevremovac”, Faculty of Biology, University of Belgrade, Takovska 43, 11000 Belgrade, Serbia; 4 Center of Plant Biotechnology and Conservation (CPBC), Takovska 43, 11000 Belgrade, Serbia

**Keywords:** *Arabidopsis thaliana*, cytokinin, expression profiles, *meta*-topolin, thidiazuron, *trans*-zeatin, temporal dynamics, ten-day-old seedlings

## Abstract

Cytokinins (CKs) are a class of phytohormones that regulate a wide range of developmental processes in plants. Different structural forms of these phytohormones have been widely used in plant tissue culture, although comparative studies on their temporal effects on gene expression remain limited. In this study, we compared the effects of *trans*-zeatin (*t*Z), *meta*-topolin (*m*T), and thidiazuron (TDZ) on the expression dynamics of ten CK-responsive genes associated with shoot growth and development in *Arabidopsis thaliana*. Ten-day-old *Arabidopsis* seedlings were exposed to liquid nutrient media containing 5 μM *t*Z, *m*T, or TDZ, or a control medium, for 15 min and 1, 2, 4, 8, and 24 h. Gene expression was analyzed by RT-qPCR using three independent biological replicates. All tested genes were strongly upregulated at 4 h following *m*T treatment, whereas most genes showed the highest expression at 8 h in response to TDZ. In contrast, *t*Z generally induced weaker and slower transcriptional responses. Expression levels of all analyzed genes returned to control-like or lower levels within 24 h. Overall, the three structurally distinct cytokinins induced markedly different temporal transcriptional responses, with *m*T producing the fastest and most pronounced effects on the expression of the tested genes under the applied experimental conditions. These findings improve our understanding of CK-dependent transcriptional dynamics during shoot development and provide a basis for future studies on CK-regulated morphogenesis and plant regeneration.

## Simple Summary

Various forms of the plant hormone cytokinin are commonly used in plant biotechnology, but the subtle differences among their effects on plant growth and development have not been comprehensively evaluated. In this work, we used the model plant *Arabidopsis thaliana* to evaluate how three different cytokinins (*trans*-zeatin, *meta*-topolin, and thidiazuron) affect the expression of ten genes related to shoot growth and development in ten-day-old *Arabidopsis* seedlings exposed to the tested cytokinins for various durations of time (15 min, 1, 2, 4, 8, and 24 h). Our results showed that *meta*-topolin generally induced a faster and more pronounced response in the expression of most of the tested genes, compared to *trans*-zeatin and thidiazuron. The dynamic response of the tested genes to various cytokinins indicates substantial indirect effects of the tested cytokinins on plant gene expression. The findings of our study represent a valuable basis for further research into the specific effects of each of the cytokinin molecules used in plant biotechnology.

## 1. Introduction

**Figure 1:**
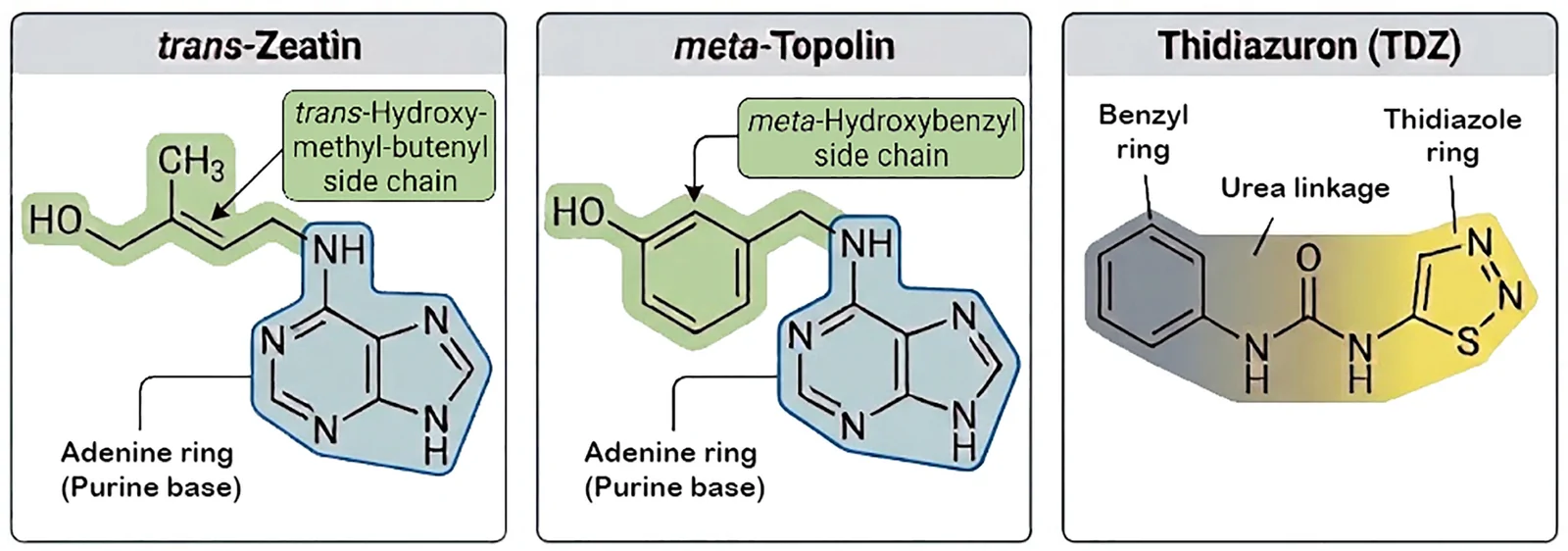
Structural features of representative isoprenoid (*trans*-zeatin, *t*Z), aromatic (*meta*-topolin, *m*T), and phenylurea-type cytokinins (thidiazuron, TDZ). Note that both isoprenoid and aromatic cytokinins consist of an adenine ring, and an isoprenoid or aromatic side chain. The *trans*-conformation of the hydroxyl group in the isoprenoid side chain of *t*Z makes it structurally analogous to the *meta*-hydroxylated aromatic side chain in *m*T. On the other hand, the molecular structure of TDZ as a phenylurea-type cytokinin is different and consists of a urea core, disubstituted with a benzyl ring and a thidiazole ring.

Cytokinins (CKs), one of the major classes of plant hormones, are involved in several developmental processes, including cell division and proliferation, callus differentiation, embryogenesis, maintenance of root and shoot meristems, vascular development, apical dominance, senescence, and morphogenesis [1,2]. Plant-derived cytokinins are derivatives of *N*^6^-substituted adenine and, depending on the chemical nature of the substituent, are classified as isoprenoid or aromatic CKs [3]. Isoprenoid cytokinins are the most common type, with *trans*-zeatin (*t*Z) being the most abundant and biologically potent in *Arabidopsis thaliana* [4,5]. Aromatic cytokinins have a benzyl ring at the *N*^6^ position; these include 6-benzyladenine (BA) [6] and *meta*-topolin (*m*T), which is considered a structural analog of *t*Z among the aromatic CKs [7] and has been increasingly used for biotechnological purposes due to its high biological activity and because it rarely causes side effects [8,9,10,11]. Synthetic cytokinins are structural derivatives of phenylurea, among which thidiazuron (TDZ) has shown strong biological activity and is widely used in in vitro culture [12,13] (Figure 1).

Upon cytokinin perception by histidine kinase receptors (AHKs), the signal is transmitted through a multistep phosphorelay involving *Arabidopsis* histidine phosphotransfer proteins (AHPs) and *Arabidopsis* response regulators (ARRs) [14,15]. Activated type-B ARRs function as transcription factors that induce the expression of primary CK-responsive genes, including type-A ARRs, thereby establishing a transcriptional regulatory network controlling shoot apical meristem activity, cell proliferation, and organ development [14,15]. Consequently, differences in receptor activation, ligand affinity, and downstream signaling properties among structurally distinct cytokinins may contribute to differences in the magnitude and timing of CK-responsive gene expression. Different cytokinin types are known to bind with varying affinities to different cytokinin receptors in *Arabidopsis*: *t*Z has the highest affinity for the receptors ARABIDOPSIS HISTIDINE KINASE3 and 4 (AHK3 and AHK4); meanwhile, TDZ binds to these receptors with moderate affinity, whereas BA exhibits low affinity [16]. Similar findings have been reported in potato (*Solanum tuberosum* L.), where TDZ and *t*Z showed high affinity for StHK receptors, while BA affinity was lower [17]. It has been suggested that the differences in binding affinities for cytokinin receptors may underlie the differences in the physiological effects of different cytokinins, including effects on gene expression [18].

Although isoprenoid, aromatic, and phenylurea-type cytokinins all activate CK signaling, previous studies have demonstrated important differences in their biological properties [14,15]. As a naturally occurring isoprenoid cytokinin, *t*Z is subject to relatively rapid metabolic turnover, whereas aromatic cytokinins, including *m*T, generally exhibit greater metabolic stability [19,20]. TDZ is structurally unrelated to naturally occurring cytokinins and has been reported to exhibit exceptionally high cytokinin activity, partly because of its persistence in plant tissues and its ability to influence endogenous CK metabolism [21,22]. In addition, differences in receptor-binding affinity among individual cytokinins have been reported, suggesting that the magnitude and timing of downstream transcriptional responses may vary depending on the applied cytokinin [14,16,23].

Numerous studies have examined the roles of different cytokinin types in regeneration processes across various plant species, primarily focusing on their effects on the proliferation and growth of plant cultures. Despite the well-documented differences in their morphogenic effects in vitro, comparative studies examining the transcriptional responses induced by different cytokinin types remain relatively scarce and have mainly emphasized similarities rather than differences [18]. The pioneering transcriptomic study by Rashotte et al. [24] has considerably expanded our understanding of CK-responsive genes at the genome-wide level. This study aimed at identifying CK-responsive targets common to *t*Z and BA at the whole-genome level and showed considerable overlap between the transcriptomic profiles of the responses to the two different cytokinins, but the two transcriptomic profiles were not identical. In a proteomic study, Černý et al. demonstrated common features, but also differences between the proteomic profiles of *t*Z-, iP-, BA- and TDZ-treated *Arabidopsis* seedlings [25]. More recently, Király et al. investigated the differences in transcriptomic responses of apple shoots to two aromatic cytokinins, *m*T and BA, applied over four weeks of in vitro culture [26].

In a previously published study from our research group, expression profiles of different shoot organogenesis-related genes during in vitro shoot regeneration of kohlrabi (*Brassica oleracea* L. var. *gongylodes*) under different CK treatments were reported [27]. Despite providing thorough insights into the activity of these genes under treatment with different cytokinin types, the study focused on long-term (≥7 days) cytokinin effects and, therefore, differences in the dynamics of the early expression responses of particular genes upon exposure to different CK signals remain unknown. Moreover, the answer to such a fundamental research question requires the use of a widely accepted model plant such as *Arabidopsis thaliana*.

Since previous studies have shown that different cytokinin types exert similar, but not identical, effects on gene expression in *Arabidopsis* [24,25], we hypothesized that structurally distinct cytokinins would differentially regulate the temporal expression of CK-responsive genes, resulting in differences in both the timing and magnitude of transcriptional responses. Despite the well-recognized differences in the biological properties of natural and synthetic cytokinins, comparative analyses of their early transcriptional effects under identical experimental conditions remain limited. In particular, little is known about how *trans*-zeatin (*t*Z), *meta*-topolin (*m*T), and thidiazuron (TDZ) differ in the temporal regulation of genes involved in shoot apical meristem maintenance, organogenesis, auxin transport, and cell cycle progression.

To address this knowledge gap, we analyzed the expression dynamics of ten CK-responsive genes following treatment with *t*Z, *m*T, and TDZ at 15 min and 1, 2, 4, 8, and 24 h under identical experimental conditions. To capture complementary aspects of CK-regulated shoot development, genes representing distinct biological processes were selected, including CK signaling (*ARABIDOPSIS RESPONSE REGULATOR5—ARR5*) [28,29], shoot apical meristem maintenance (*SHOOTMERISTEMLESS*—*STM* and *WUSCHEL—WUS*) [30,31,32,33], organ boundary establishment (*CUP-SHAPED COTYLEDON1—CUC1* and *ROOT GROWTH-DEFECTIVE3*—*RGD3*) [34,35], vascular patterning and auxin transport (*PIN-FORMED—PIN3* and *PIN4*) [36], radial patterning (*SHORTROOT—SHR*) [37], and cell cycle regulation (*CYCLIN B2;4—CYCB2;4* and *CYCLIN-DEPENDENT KINASE B2;1—CDKB2;1*) [38,39]. Together, these genes provide a representative framework for evaluating the temporal effects of structurally distinct cytokinins on transcriptional networks underlying shoot development [27,37,40,41]. Although they do not encompass the entire CK-responsive transcriptome, they were selected to represent major functional modules of CK-regulated shoot development while enabling a detailed temporal analysis of genes with well-established developmental roles.

The objective of this study was therefore to compare the temporal expression dynamics of these representative CK-responsive genes following treatment of 10-day-old *Arabidopsis* seedlings with 5 μM *t*Z, *m*T, and TDZ for 15 min or 1, 2, 4, 8, or 24 h under identical experimental conditions and to determine whether structurally distinct cytokinins elicit different transcriptional response patterns in terms of response timing and magnitude.

## 2. Materials and Methods

### 2.1. Plant Material

#### 2.1.1. Seed Sterilization

Seeds of *A*. *thaliana* Col-0 were surface-sterilized using 70% ethanol and freshly prepared sodium hypochlorite solution. The seeds were first immersed in 70% ethanol for 30 s, after which they were treated for 15 min with a 2.5% (*v*/*v*) NaOCl solution prepared by diluting equal volumes of commercial bleach containing 5% NaOCl with sterile distilled water, supplemented with 50 μL of the non-ionic surfactant Tween^®^ 20 (SERVA Electrophoresis GmbH, Heidelberg, Germany). Approximately 1000 seeds were sterilized in 10 mL of the sterilization solution. The seeds were subsequently washed eight times with 1 mL of sterile distilled water to remove residual NaOCl. After sterilization, the seeds were aseptically stored in the dark at 4 °C for four days (cold stratification). Seed sterility was monitored throughout germination and subsequent seedling cultivation, and no microbial contamination was observed. Germination reached approximately 85%, indicating that the sterilization procedure effectively eliminated microbial contamination without adversely affecting seed viability.

#### 2.1.2. Seedling Germination and Growth

All seeds were germinated on a basal medium under aseptic conditions at a density of eight seeds per Petri plate. The basal medium consisted of Murashige and Skoog (MS) mineral salts [42], Linsmaier and Skoog vitamins [43], 100 mg L^−1^ *myo*-inositol, 3% sucrose, and 0.7% agar. The pH of the medium was adjusted to 5.8 with 1 N NaOH and sterilized by autoclaving at 114 °C and 80 kPa for 25 min according to the validated standard operating procedure routinely used in our laboratory. Complete medium sterility was consistently achieved under these conditions, and no microbial contamination was observed during the experiments. Seedlings were cultured in vitro in a vertical position in a growth chamber under continuous white LED illumination (80 ± 5 μmol m^−2^ s^−1^; PG200N lights, Center of LED and Optoelectronic Technologies, Minsk, Belarus) at 23 ± 2 °C until 10 days after germination (DAG). Germination reached approximately 85%, and only healthy, uniformly developed seedlings were selected for cytokinin treatments. Ten-day-old seedlings were chosen because, at this developmental stage, they exhibit actively developing shoot tissues while maintaining a high degree of developmental uniformity among biological replicates [24].

#### 2.1.3. CK Treatment of Seedlings and Sample Collection

**Figure 2:**
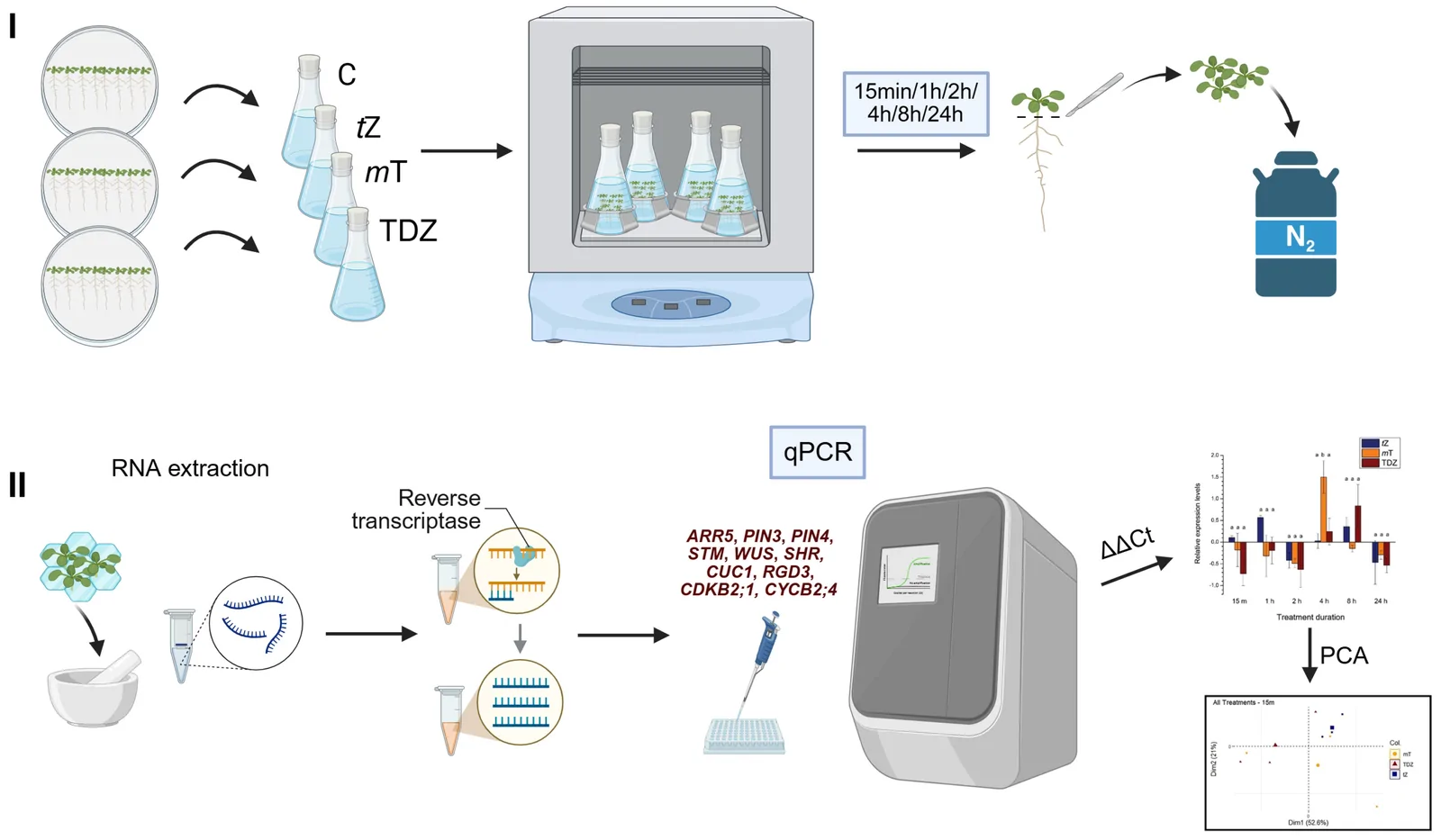
Overview of the experimental workflow for treating 10-day-old *Arabidopsis* seedlings with different cytokinins. (**I**) Seedlings grown under controlled conditions and constant lighting were transferred to media containing *t*Z, *m*T, or TDZ, or a hormone-free medium, followed by incubation for 15 min and 1, 2, 4, 8, and 24 h on an orbital shaker. Shoots from treated seedlings were flash-frozen in liquid nitrogen and stored at −80 °C. (**II**) RNA was extracted from the collected samples and used for reverse transcription, followed by quantitative PCR (qPCR) for gene expression analysis. Expression levels were normalized using the ΔΔCt method, and Principal Component Analysis (PCA) was applied to assess the variability in the measured parameters under the different CK treatments (*t*Z, *m*T, and TDZ) across the six time points (15 min, 1, 2, 4, 8, and 24 h). The arrows between parts of the figure represent the sequence of steps performed in the workflow. The figure was created in BioRender (Cosic, T. (2026); https://BioRender.com/lrpssrd; accessed on 7 August 2026).

After 10 days of growth under the conditions described above, intact seedlings were harvested from the plates and transferred to liquid MS medium supplemented with different cytokinins. The liquid MS medium was prepared as described above but without agar, and dispensed into 100 mL Erlenmeyer flasks, each containing 50 mL of medium supplemented with 5 μM *t*Z, *m*T, or TDZ (OlChemIm s.r.o., Olomouc, Czech Republic). The control consisted of liquid MS medium without cytokinins. The applied cytokinin concentration (5 μM) was selected based on the experimental design of Rashotte et al. [24], in which this concentration proved suitable for investigating CK-induced transcriptional responses in *Arabidopsis*. This concentration has subsequently been adopted as a standard concentration in transcriptomic and proteomic CK-response studies [24,25,26]. The experimental design was based on the approach described by Rashotte et al. [24], adapted for the comparison of three structurally distinct cytokinins (*t*Z, *m*T, and TDZ). To minimize the influence of individual plant variation, seedlings were selected for different treatments by randomly choosing every second, fourth, fifth, or seventh seedling from Petri dishes containing eight seedlings each. Fourteen uniformly grown seedlings were transferred into each Erlenmeyer flask and incubated on an orbital shaker (SBM-X, Adolf Kühner AG, Birsfelden, Switzerland) at 95 rpm for 15 min or 1, 2, 4, 8, or 24 h, according to [24]. The treatments were initiated at different times of day so that all the samples could be collected at the same time of day (between 5 and 6 PM) to minimize the potential influence of circadian regulation on CK-responsive gene expression. Shoots were collected under aseptic conditions by separating them from the roots, flash-frozen in liquid nitrogen, and stored at −80 °C (Figure 2). Each biological replicate consisted of pooled shoots from 14 seedlings to ensure sufficient high-quality RNA for downstream qRT-PCR analysis. The entire experiment was performed using three independent biological replicates, each comprising the pooled shoots of 14 seedlings incubated together in a single Erlenmeyer flask.

### 2.2. qRT-PCR Analysis

#### 2.2.1. RNA Isolation

Total RNA extraction from the frozen control and CK-treated samples was performed according to the CTAB-based protocol of Gašić et al. [44], with the modifications described by Ćosić et al. [27], including optimization of the extraction procedure for CK-treated shoot tissues. Briefly, RNA was extracted from 100–150 mg of frozen shoot tissue using a series of chloroform:isoamyl alcohol (24:1, *v*/*v*) extractions, followed by precipitation with 7.5 M LiCl and resuspension in RNase-free water. RNA concentration and purity were assessed spectrophotometrically using a Implen Nanophotometer N60 NanoDrop instrument (Implen GmbH, Munich, Germany) by measuring the A_260_/A_280_ absorbance ratio, while RNA integrity was verified by agarose gel electrophoresis. Only RNA samples showing appropriate purity and integrity were used for downstream analyses.

#### 2.2.2. Reverse Transcription and Quantitative Real-Time PCR

DNase I (Thermo Scientific, Waltham, MA, USA) treatment was performed on 2 μg of total RNA at 37 °C for 30 min to eliminate residual genomic DNA prior to reverse transcription. Reverse transcription of RNA to cDNA was carried out using RevertAid™ reverse transcriptase and random hexamer primers (Thermo Scientific). The reactions were set up according to the manufacturer’s instructions. The reaction components and conditions are described in detail in our previous work [27]. Reverse transcription was performed using an Eppendorf™ Mastercycler™ Nexus Thermal Cycler (Eppendorf, Hamburg, Germany).

The obtained cDNA was used for qPCR using a QuantStudio™ 3 Real-Time PCR System (Thermo Scientific); three independent biological replicates were tested, each with technical duplicates. The reactions were performed with SYBR Green I (Maxima SYBR Green/ROX Kit, Thermo Scientific) according to the manufacturer’s protocol. Each 10 μL qPCR reaction contained 1 μL of cDNA (20 ng), 5 μL of qRT-PCR Master Mix, and 0.6 μL of 5 μM forward and reverse primers for each of the eleven genes (Table 1). All primers were designed using the NCBI’s Primer-BLAST software tool (version 2.5.0). The specificity of the primers was verified via BLAST, by blotting the RT-PCR products onto agarose gels, and by melting curve analysis to verify the absence of non-specific products and primer dimers. The conditions for the qPCR reaction were set as: initial denaturation (95 °C, 5 min); 40 cycles (denaturation at 95 °C, 30 s; annealing at 60 °C, 1 min; extension at 72 °C, 1 min); final extension (72 °C, 10 min), after which melting curve analysis was performed by cooling down to 60 °C and monitoring the fluorescence of the qPCR reactions until the final temperature of 95 °C was achieved at a slope of 0.1 °C s^−1^ for 6 min [27].

To normalize the expression levels of the target genes, actin (*ACT7*; Table 1) was used as the reference gene [45,46]. Using the ΔΔCt method, the expression levels were calculated relative to the expression in the corresponding untreated controls at each time point [47]. Relative expression levels were calculated as log_2_-transformed fold changes.

The efficiency of the qPCR reactions for each analyzed gene was examined using standards prepared from cDNA obtained from the RT reaction, as described in detail in [27]. The R^2^ values ranged from 0.991 to 0.999, values for amplification efficiency ranged from 93.6% to 101.6%, and slope ranged from −3.284 to −3.484.

### 2.3. Data Analysis

#### 2.3.1. Statistical Analyses

Statistical analysis of the qRT-PCR results was performed to evaluate the effects of different treatments (control, *t*Z, *m*T, and TDZ) on relative gene expression levels (log_2_R values) across six distinct time points (15 min, 1 h, 2 h, 4 h, 8 h, and 24 h). All relative expression results were included in the statistical analyses, with the exception of *CLAVATA3* (*CLV3*), for which no significant expression was detected due to technical limitations, as explained in the Section 3.1. To ensure accurate variance estimation, all three biological replicates per experimental group were treated as independent experimental units in the statistical models. Before conducting the analysis of variance (ANOVA), the underlying parametric assumptions were systematically validated for each gene and time point. Normality of distribution was formally evaluated within each individual experimental group using the Shapiro–Wilk test (*n* = 3). Homogeneity of variances across treatment groups within each specific time point was assessed using Levene’s test based on the median, which is robust against minor deviations. The datasets successfully satisfied both prerequisites, displaying non-significant deviations from normality and equal variances (*p* > 0.05). Following assumption validation, a One-Way ANOVA was executed for each time point to detect significant differences among the treatments, and post-hoc multiple comparisons were conducted using Fisher’s Least Significant Difference (LSD) test at the 5% level of probability (*p* ≤ 0.05) to evaluate differences between means, consistently with previous similar studies [27,48,49]. To address the potential inflation of Type I error risk arising from analyzing a large number of genes and time points, and to ensure statistical robustness beyond simple *p*-value thresholds, the effect size was explicitly calculated. The magnitude of the treatment effect was quantified using Partial Eta Squared (η_p_^2^), defined as:

η_p_^2^ = SS_treatment_/(SS_treatment_ + SS_residual_)

where SS_treatment_ is the effect sum of squares and SS_residual_ is the error sum of squares. Values of η_p_^2^ ≥ 0.14 were interpreted as a large effect size, providing additional confidence in the biological relevance of the observed changes. The main One-Way ANOVA modeling and subsequent post-hoc comparisons (Fisher’s LSD test) were executed using SAS software (SAS/STAT, version 9.00; SAS Institute Inc., Cary, NC, USA). Preliminary testing of ANOVA assumptions (Shapiro–Wilk and Levene’s tests) and calculation of effect sizes (η_p_^2^) were performed computationally using the Python software environment (version 3.10). The complete validation matrices and effect size values are compiled in Supplementary Table S1.

#### 2.3.2. Principal Component Analysis (PCA)

Principal Component Analysis (PCA) was performed on the remaining 10 tested genes using their relative expression levels as input variables. The FactoMineR package in R (version 4.2.2) was used to perform the PCA [50] and the Factoextra package was used to visualize the data distributions [51]. The contribution of individual genes to the principal components was calculated to identify the variables driving sample separation. To statistically evaluate differences among sampling time points in the multivariate gene expression space, permutational multivariate analysis of variance (PERMANOVA) was performed in R using the vegan package [52] according to the method described by [53], with 999 permutations.

## 3. Results

### 3.1. Gene Expression Changes in Response to Different CK Treatments

Quantitative Real-Time PCR was used to amplify the genes *ARR5*, *PIN3*, *PIN4*, *STM*, *WUS*, *CLV3*, *CUC1*, *RGD3*, *SHR*, *CYCB2;4*, and *CDKB2;1*, and to determine their expression levels.

The qRT-PCR amplification of *CLV3* was very low in all samples. As *CLV3* expression is normally restricted to the apex of the SAM [54,55], *CLV3* mRNA accounts for a very small fraction of the total RNA when whole shoots are used for RNA extraction, which is likely the reason for its weak amplification. We conclude, based on this result, that *CLV3* does not appear to be a suitable gene for expression studies on tissue samples comparable in size to whole shoots of *Arabidopsis* seedlings.

The remaining ten genes showed significant upregulation induced by *m*T at the 4 h time point, while the gene expression responses to *t*Z and TDZ differed, with most genes upregulated by TDZ at 8 h.

**Figure 3:**
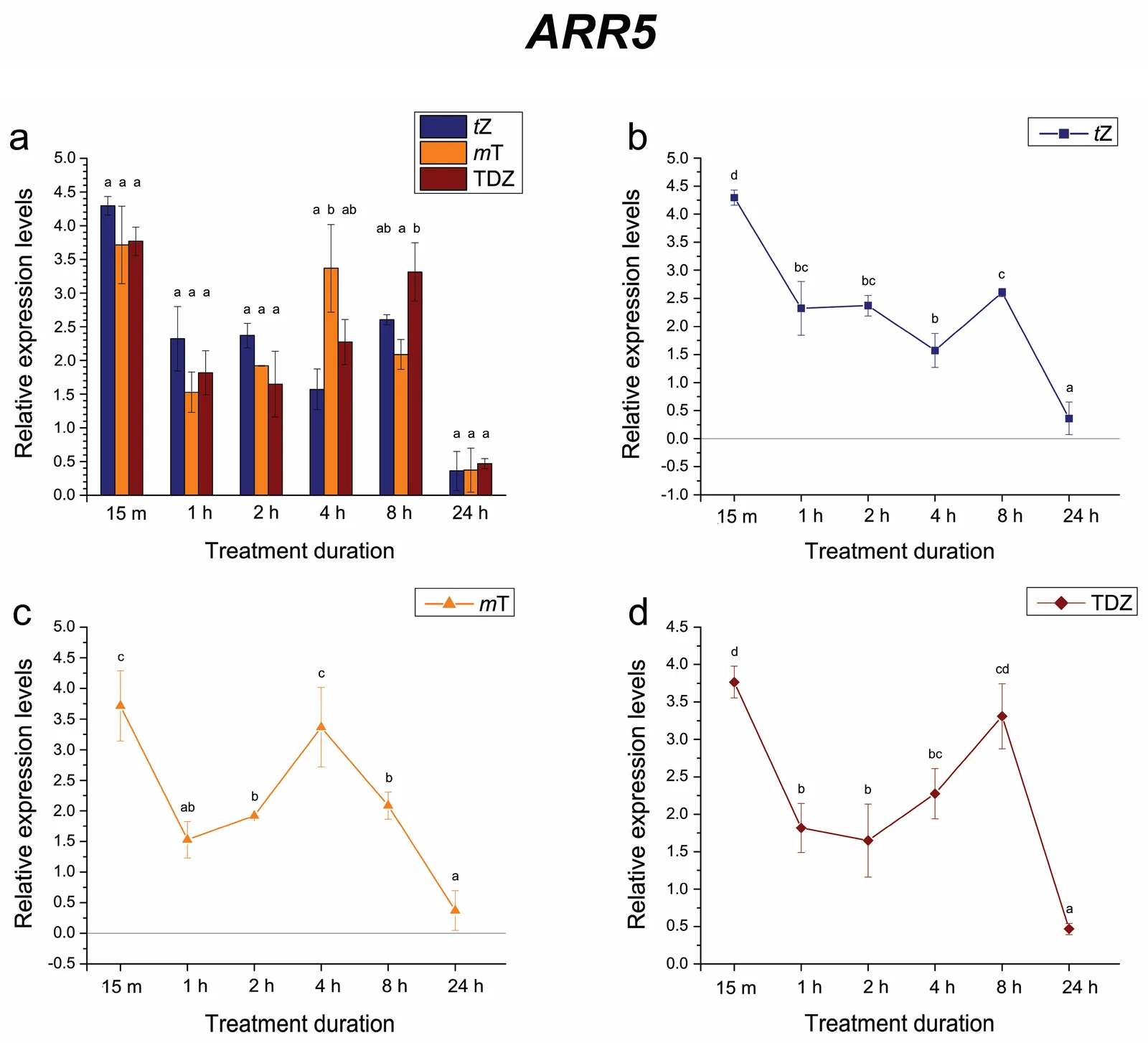
Expression dynamics of *ARR5* in 10-day-old *Arabidopsis* seedlings during 24 h of CK exposure. (**a**) Comparison of relative expression levels of *ARR5* in 10-day-old *Arabidopsis* seedlings during 24 h of exposure to *t*Z, *m*T, and TDZ. For each time point, means with the same letter are not significantly different between the CK treatments according to the LSD post-hoc test (*p* ≤ 0.05). (**b**–**d**) Relative expression levels of *ARR5* in 10-day-old *Arabidopsis* seedlings during 24 h of exposure to *t*Z (**b**), *m*T (**c**), and TDZ (**d**). Means with the same letter within each graph are not significantly different according to the LSD post-hoc test (*p* ≤ 0.05). All relative expression levels were calculated using the ΔΔCt method relative to untreated controls at each time point and are expressed as log_2_ values. The relative expression level in the control equals log_2_ Ct/Ct = 0 for each of the time points. All results are presented as mean values with corresponding standard errors based on three independent biological replicates. *t*Z: *trans*-zeatin; *m*T: *meta*-topolin; TDZ: thidiazuron.

*ARR5* expression differed from the other genes and showed sustained upregulation over time (Figure 3a). All treatments showed rapid increases in expression at 15 min, followed by a decline and a second peak at 4–8 h, before decreasing at 24 h. In *t*Z-treated samples, expression peaked at 15 min and slightly increased again at 8 h (Figure 3b). *m*T induced peaks at 15 min and 4 h (Figure 3c), while TDZ produced peaks at 15 min and 8 h, both followed by a decline at 24 h (Figure 3d).

**Figure 4:**
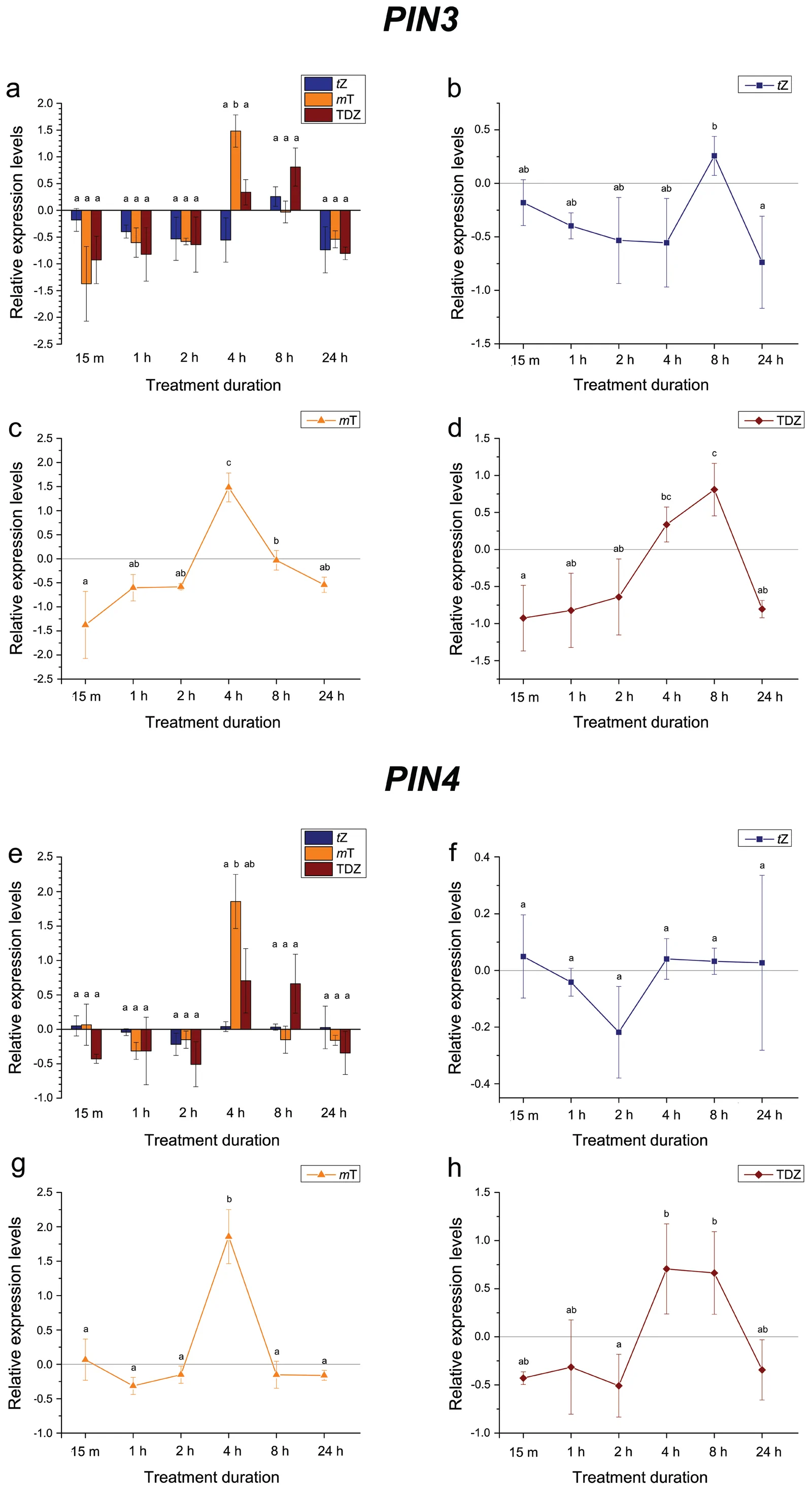
Expression dynamics of *PIN3* and *PIN4* in 10-day-old *Arabidopsis* seedlings during 24 h of CK exposure. (**a**) Comparison of relative expression levels of *PIN3* in 10-day-old *Arabidopsis* seedlings during 24 h of exposure to *t*Z, *m*T, and TDZ. (**b**–**d**) Relative expression levels of *PIN3* in 10-day-old *Arabidopsis* seedlings during 24 h of exposure to *t*Z (**b**), *m*T (**c**), and TDZ (**d**). (**e**) Comparison of relative expression levels of *PIN4* in 10-day-old *Arabidopsis* seedlings during 24 h of exposure to *t*Z, *m*T and TDZ. (**f**–**h**) Relative expression levels of *PIN4* in 10-day-old *Arabidopsis* seedlings during 24 h of exposure to *t*Z (**f**), *m*T (**g**), and TDZ (**h**). According to the LSD post-hoc test (*p* ≤ 0.05), means with the same letter within the same time point (**a**,**e**) or graph (**b**–**d**,**f**–**h**) are not significantly different between the CK treatments or time points, respectively. All relative expression levels were calculated using the ΔΔCt method relative to untreated controls at each time point and are expressed as log_2_ values. The relative expression level in the control equals log_2_ Ct/Ct = 0 for each of the time points. All results are presented as mean values with corresponding standard errors based on three independent biological replicates. *t*Z: *trans*-zeatin; *m*T: *meta*-topolin; TDZ: thidiazuron.

The expression profiles of *PIN3* and *PIN4* showed similar patterns, with both genes slightly downregulated at the early time points with all three CK treatments (Figure 4a,e). This initial downregulation was followed by a prominent upregulation at 4 h in *m*T-treated samples (Figure 4c,g). Both genes were also upregulated after 4 h in TDZ-treated samples, though the increases were less pronounced than with *m*T (Figure 4d,h). In contrast, *t*Z did not induce significant expression changes, except for a slight upregulation of *PIN3* at 8 h (Figure 4b).

**Figure 5:**
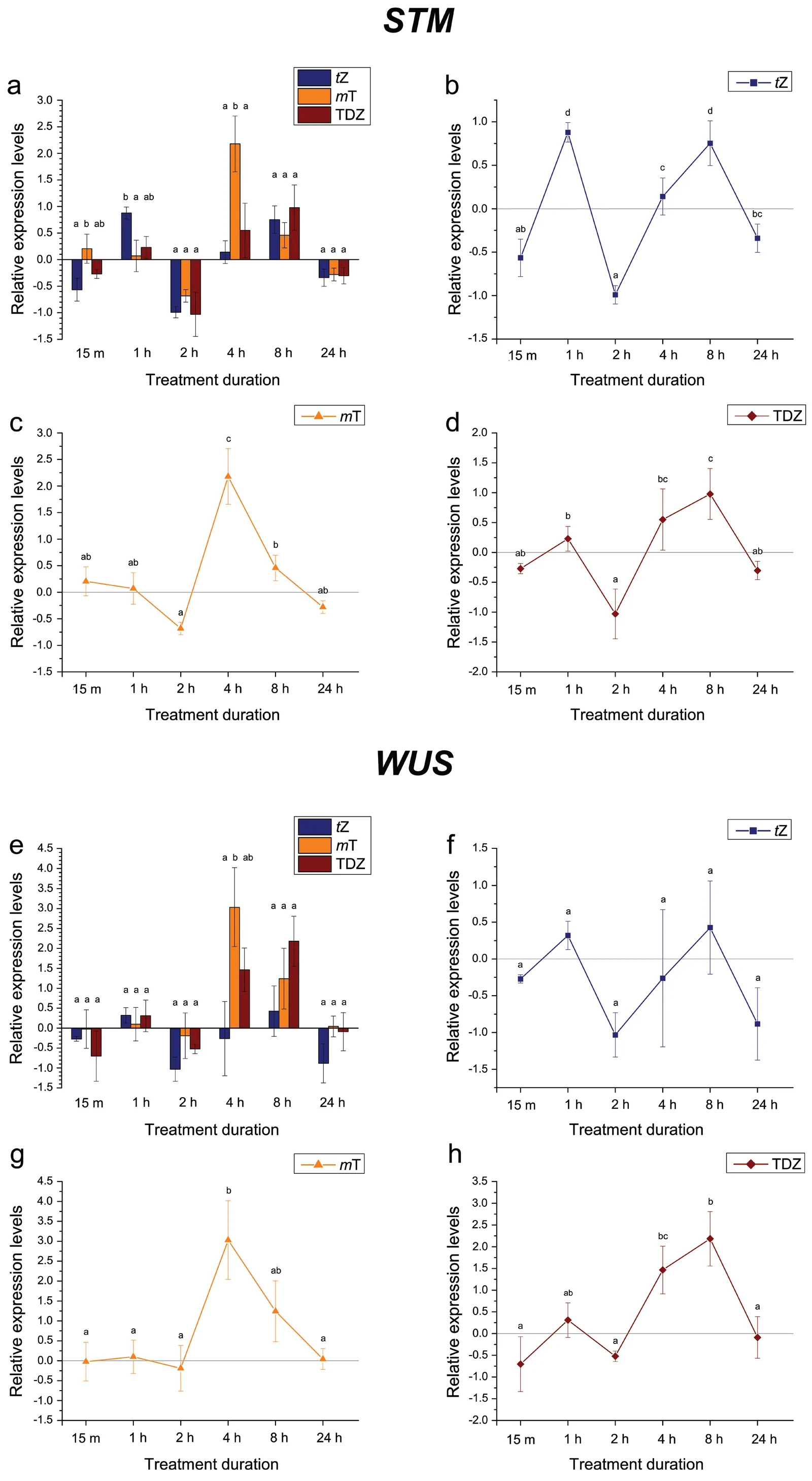
Expression dynamics of *STM* and *WUS* in 10-day-old *Arabidopsis* seedlings during 24 h of CK exposure. (**a**) Comparison of relative expression levels of *STM* in 10-day-old *Arabidopsis* seedlings during 24 h of exposure to *t*Z, *m*T, and TDZ. (**b**–**d**) Relative expression levels of *STM* in 10-day-old *Arabidopsis* seedlings during 24 h of exposure to *t*Z (**b**), *m*T (**c**), and TDZ (**d**). (**e**) Comparison of relative expression levels of *WUS* in 10-day-old *Arabidopsis* seedlings during 24 h of exposure to *t*Z, *m*T and TDZ. (**f**–**h**) Relative expression levels of *WUS* in 10-day-old *Arabidopsis* seedlings during 24 h of exposure to *t*Z (**f**), *m*T (**g**), and TDZ (**h**). According to the LSD post-hoc test (*p* ≤ 0.05), means with the same letter within each time point (**a**,**e**) or within each graph (**b**–**d**,**f**–**h**) are not significantly different between the CK treatments or time points, respectively. All relative expression levels were calculated using the ΔΔCt method relative to untreated controls at each time point and are expressed as log_2_ values. The relative expression level in the control equals log_2_ Ct/Ct = 0 for each of the time points. All results are presented as mean values with corresponding standard errors based on three independent biological replicates. *t*Z: *trans*-zeatin; *m*T: *meta*-topolin; TDZ: thidiazuron.

*STM* and *WUS* exhibited comparable increases and decreases in expression over time, with both genes displaying fluctuating expression, with a significant increase induced by *m*T at 4 h (Figure 5a,e). With *t*Z treatment, *STM* expression varied markedly over time, reaching its maximum at 1 and 8 h and its lowest level at 2 h (Figure 5b). TDZ induced a strong decrease at 2 h, followed by an increase in expression at 4 and 8 h (Figure 5d). However, *WUS* was not significantly affected by TDZ treatment at the early time points, with upregulation observed at 4 and 8 h (Figure 5h).

**Figure 6:**
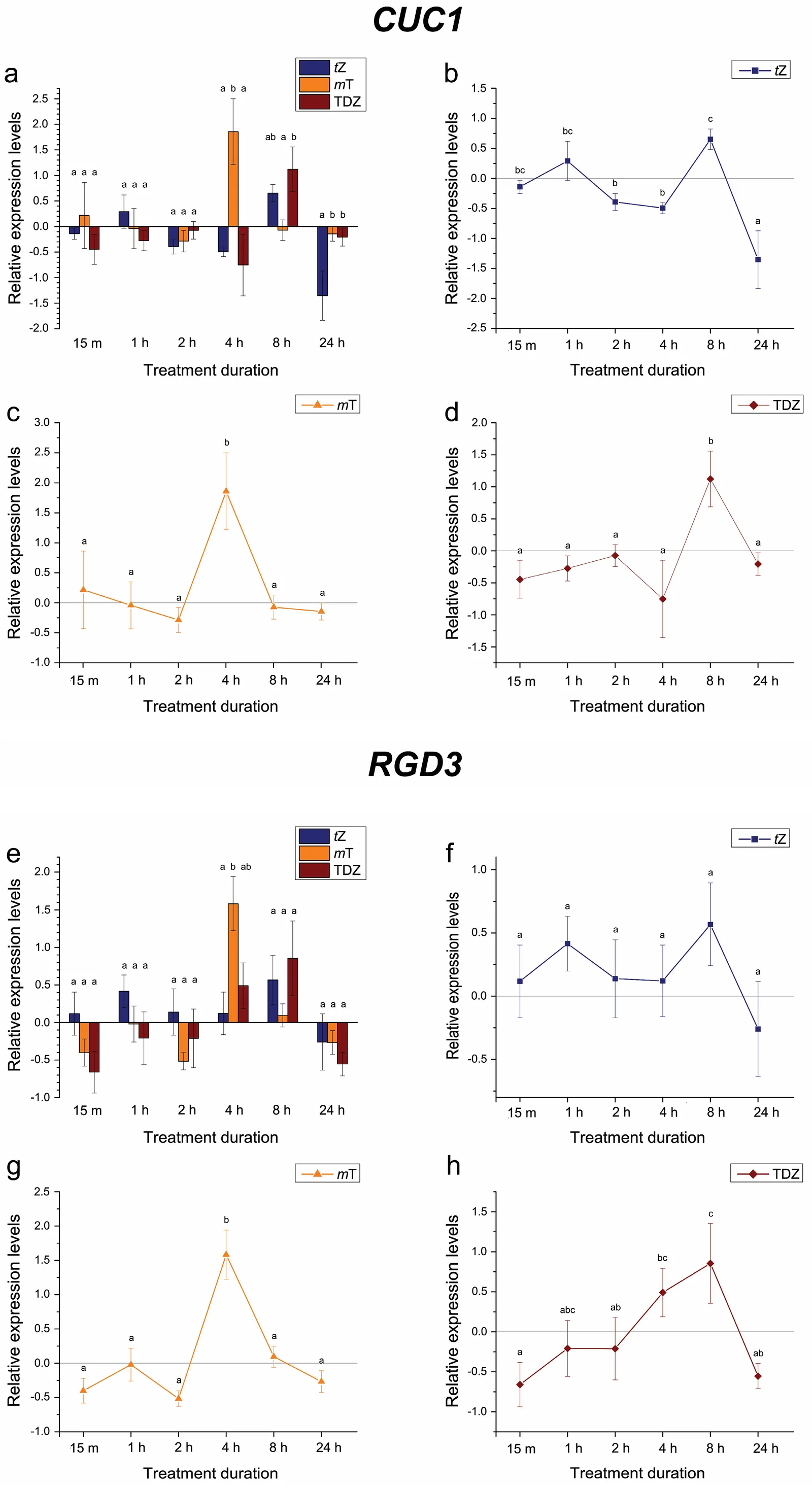
Expression dynamics of *CUC1* and *RGD3* in 10-day-old *Arabidopsis* seedlings during 24 h of CK exposure. (**a**) Comparison of relative expression levels of *CUC1* in 10-day-old *Arabidopsis* seedlings during 24 h of exposure to *t*Z, *m*T, and TDZ. (**b**–**d**) Relative expression levels of *CUC1* in 10-day-old *Arabidopsis* seedlings during 24 h of exposure to *t*Z (**b**), *m*T (**c**), and TDZ (**d**). (**e**) Comparison of relative expression levels of *RGD3* in 10-day-old *Arabidopsis* seedlings during 24 h of exposure to *t*Z, *m*T and TDZ. (**f**–**h**) Relative expression levels of *RGD3* in 10-day-old *Arabidopsis* seedlings during 24 h of exposure to *t*Z (**f**), *m*T (**g**), and TDZ (**h**). According to the LSD post-hoc test (*p* ≤ 0.05), means with the same letter within each time point (**a**,**e**) or within each graph (**b**–**d**,**f**–**h**) are not significantly different between the CK treatments or time points, respectively. All relative expression levels were calculated using the ΔΔCt method relative to untreated controls at each time point and are expressed as log_2_ values. The relative expression level in the control equals log_2_ Ct/Ct = 0 for each of the time points. All results are presented as mean values with corresponding standard errors based on three independent biological replicates. *t*Z: *trans*-zeatin; *m*T: *meta*-topolin; TDZ: thidiazuron.

The peak in expression at 4 h with *m*T treatment was also observed for *CUC1* (Figure 6a,c), while *t*Z and TDZ notably induced the expression of this gene at 8 h (Figure 6a,b,d). The expression of *CUC1* showed two prominent events during the time course of the *t*Z treatment: a visible upregulation at 8 h and a sharp drop in expression at 24 h (Figure 6b). A somewhat similar expression dynamic was observed for *RGD3*, where *m*T also elicited a substantial rise in expression at 4 h (Figure 6e,g), whereas TDZ induced upregulation that peaked at 8 h (Figure 6h).

**Figure 7:**
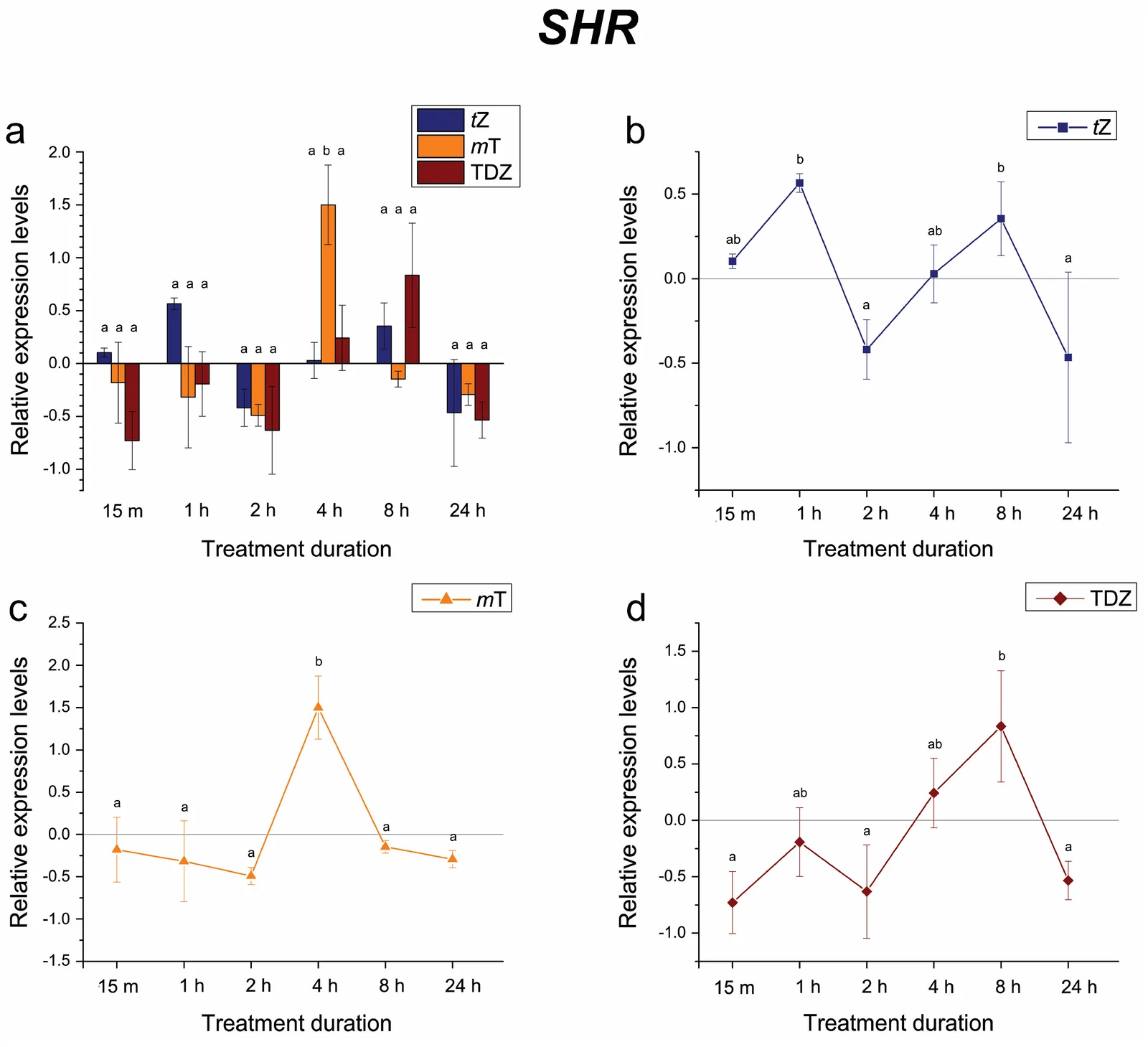
Expression dynamics of *SHR* in 10-day-old *Arabidopsis* seedlings during 24 h of CK exposure. (**a**) Comparison of relative expression levels of *SHR* in 10-day-old *Arabidopsis* seedlings during 24 h of exposure to *t*Z, *m*T, and TDZ. Within each time point, means with the same letter are not significantly different between the CK treatments according to the LSD post-hoc test (*p* ≤ 0.05). (**b**–**d**) Relative expression levels of *SHR* in 10-day-old *Arabidopsis* seedlings during 24 h of exposure to *t*Z (**b**), *m*T (**c**), and TDZ (**d**). Means with the same letter within each graph are not significantly different according to the LSD post-hoc test (*p* ≤ 0.05). All relative expression levels were calculated using the ΔΔCt method relative to untreated controls at each time point and are expressed as log_2_ values. The relative expression level in the control equals log_2_ Ct/Ct = 0 for each of the time points. All results are presented as mean values with corresponding standard errors based on three independent biological replicates. *t*Z: *trans*-zeatin; *m*T: *meta*-topolin; TDZ: thidiazuron.

Similarly to the other investigated genes, *SHR* transcript abundance was strongly elevated with *m*T treatment at 4 h (Figure 7a,c); all the other comparisons between treatments were not statistically significant (Figure 7a). In contrast, with *t*Z treatment, *SHR* transcript levels rose significantly at two time points, at 1 and 8 h, but were markedly downregulated at 2 h (Figure 7b). TDZ induced an upregulation peak at 8 h (Figure 7d).

**Figure 8:**
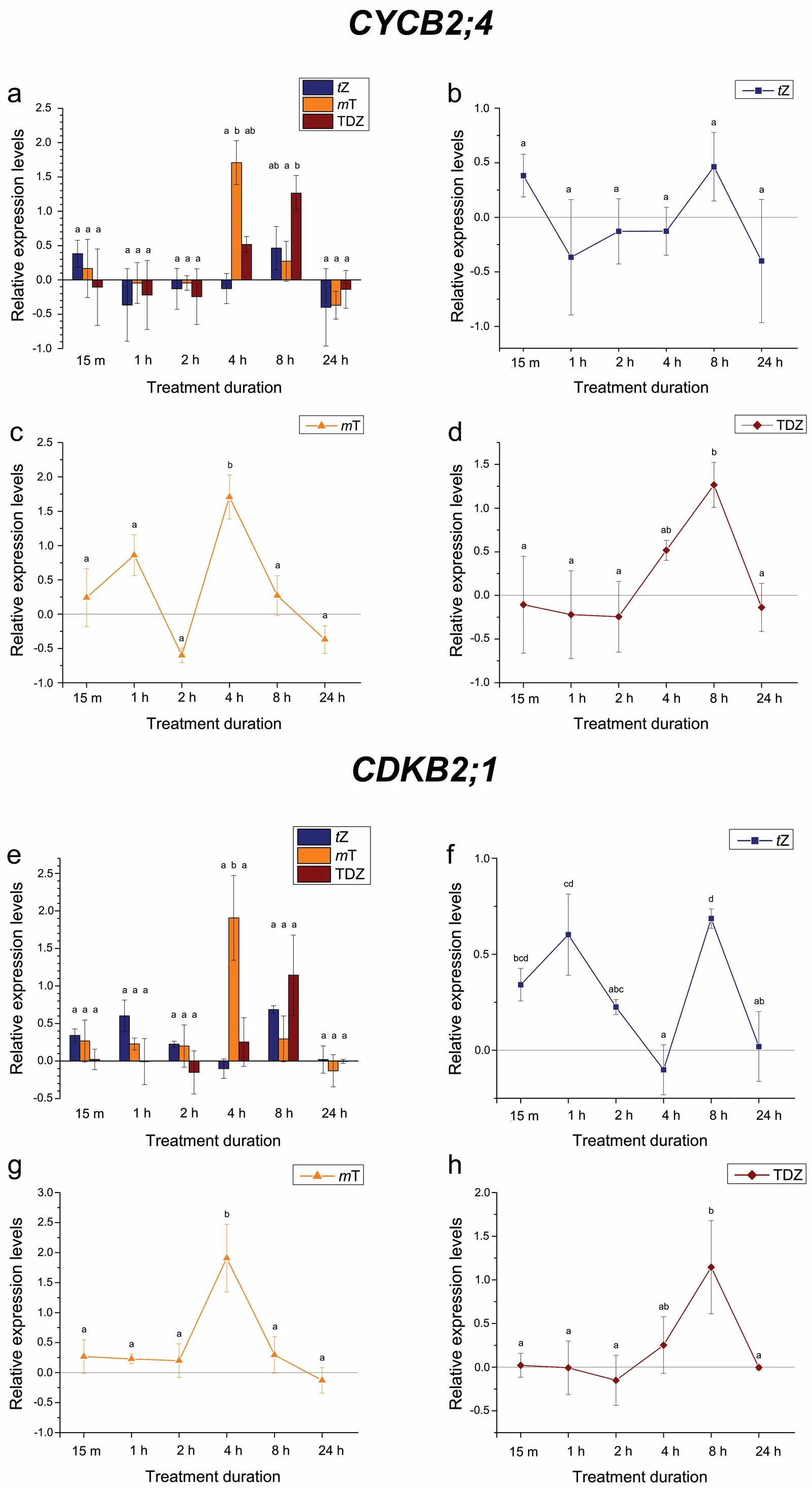
Expression dynamics of *CYCB2;4* and *CDKB2;1* in 10-day-old *Arabidopsis* seedlings during 24 h of CK exposure. (**a**) Comparison of relative expression levels of *CYCB2;4* in 10-day-old *Arabidopsis* seedlings during 24 h of exposure to *t*Z, *m*T, and TDZ. (**b**–**d**) Relative expression levels of *CYCB2;4* in 10-day-old *Arabidopsis* seedlings during 24 h of exposure to *t*Z (**b**), *m*T (**c**), and TDZ (**d**). (**e**) Comparison of relative expression levels of *CDKB2;1* in 10-day-old *Arabidopsis* seedlings during 24 h of exposure to *t*Z, *m*T, and TDZ. (**f**–**h**) Relative expression levels of *CDKB2;1* in 10-day-old *Arabidopsis* seedlings during 24 h of exposure to *t*Z (**f**), *m*T (**g**), and TDZ (**h**). According to the LSD post-hoc test (*p* ≤ 0.05), means with the same letter within each time point (**a**,**e**) or within each graph (**b**–**d**,**f**–**h**) are not significantly different between the CK treatments or time points, respectively. All relative expression levels were calculated using the ΔΔCt method relative to untreated controls at each time point and are expressed as log_2_ values. The relative expression level in the control equals log_2_ Ct/Ct = 0 for each of the time points. All results are presented as mean values with corresponding standard errors based on three independent biological replicates. *t*Z: *trans*-zeatin; *m*T: *meta*-topolin; TDZ: thidiazuron.

Two distinct expression peaks were observed for the cell-cycle genes *CYCB2;4* and *CDKB2;1*, which occurred at 4 h with *m*T and 8 h with TDZ (Figure 8a,e). The expression of *CDKB2;1* with *t*Z treatment was highly dynamic over time. Specifically, *CDKB2;1* was slightly upregulated at 15 min, which increased further at 1 h but was almost immediately followed by a sharp decrease, resulting in downregulation at 4 h. The expression level rose sharply again at 8 h, followed by an equally sudden decline at 24 h (Figure 8f). The expression profiles of both genes with *m*T treatment were similar, showing a clear peak at 4 h (Figure 8c,g).

### 3.2. Principal Component Analysis

Principal Component Analysis (PCA) was performed to assess the variability in the measured parameters under the different CK treatments (*t*Z, *m*T, and TDZ) across the six time points (15 min, 1, 2, 4, 8, and 24 h). The input parameters were the relative expression levels of the ten tested genes.

**Figure 9:**
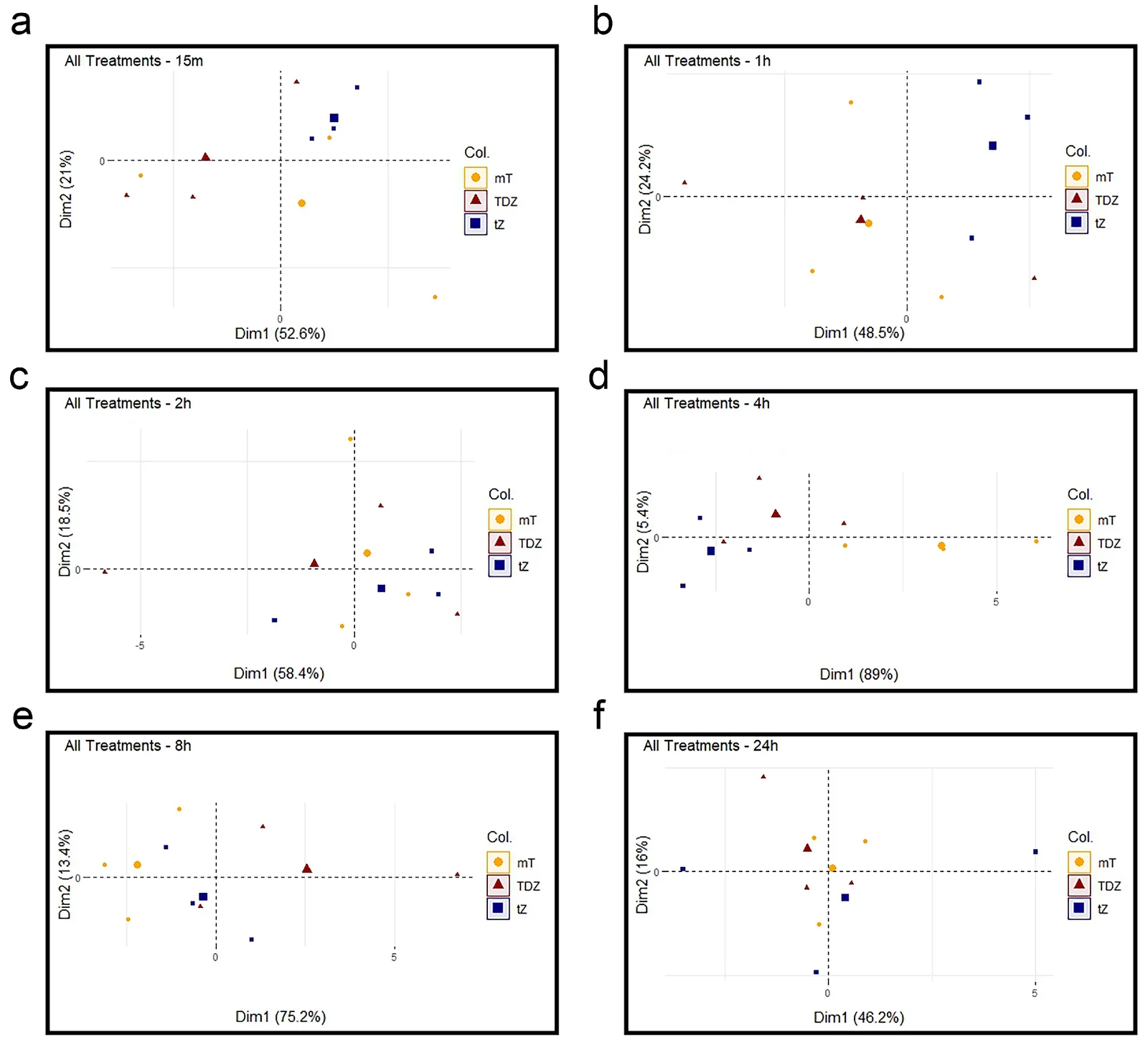
Principal Component Analysis (PCA) of the relative expression levels of the ten tested genes in 10-day-old *Arabidopsis* seedlings treated with *t*Z, *m*T, and TDZ, measured at six time points: (**a**) 15 min; (**b**) 1 h; (**c**) 2 h; (**d**) 4 h; (**e**) 8 h; (**f**) 24 h. The data distribution across the PCA matrix is represented by blue squares (data from the three replicates of the *t*Z treatment), yellow circles (*m*T treatment), and red triangles (TDZ treatment). The larger shapes indicate the centers of the data variation for each treatment. The PCA was performed using the FactoMineR package in R [50] and visualized using the FactoExtra package [51]. *t*Z: *trans*-zeatin; *m*T: *meta*-topolin; TDZ: thidiazuron.

At 15 min, the data distributions for the three CK treatments showed little overlap, with the centers of the data variations relatively well separated (Figure 9a). After 1 h, TDZ largely coincided with *m*T, while *t*Z remained somewhat shifted (Figure 9b). After 2 h of treatment, the three centers of the data variations moved closer together (Figure 9c); however, at 4 h they became notably more distant, with the *m*T data shifted along the x-axis (Figure 9d). After 8 h, the centers of the data variations were slightly closer than at 4 h, but the *m*T data clustered closer to the negative end of the x-axis rather than the positive end (Figure 9e). At the final time point (24 h), the centers were again very close, with significant data overlap (Figure 9f).

To determine whether the 4 h time point represented the strongest divergence among CK treatments in our study, PERMANOVA was performed across all analyzed time points. The highest separation among treatments was observed at 4 h (R^2^ = 0.678, F = 5.607, *p* = 0.006), confirming the PCA-based observation (Supplementary Table S2). PCA loading analysis further identified the genes contributing most strongly to treatment separation at this time point. The individual contributions of each gene to the first five dimensions of the PCA matrix are presented in Supplementary Table S3. Notably, the first PCA dimension (dim1), contributing to 89% of total data variation at 4 h, is marked by a balanced contribution of all the genes to total data variation, ranging from 7.9% for *WUS*, to 10.9% for *SHR*. By contrast, the data variation in the second and third dimension of the PCA matrix was dominated by the variation in the expression levels of both *CUC1* and *WUS* (Supplementary Table S3).

## 4. Discussion

Although the differences between the effects of particular cytokinin molecules in in vitro culture have been extensively documented in the literature (e.g., [27,56,57,58]), their choice for biotechnological use is still made on an empirical basis, regardless of mechanistic differences in their effects on the expression of particular genes. The extent to which these differences in gene expression responses occur, and the particular dynamics with which different cytokinin types induce them when exogenously applied, remain poorly characterized. In this work, we show that *t*Z, *m*T, and TDZ affect the temporal expression profiles of the 10 tested genes in *Arabidopsis* in distinct ways, with *m*T generally inducing the highest upregulation by 4 h; TDZ usually induced a somewhat weaker and more delayed upregulation, whereas *t*Z mostly showed the weakest effect on gene expression in our experimental setting.

CK responses are initiated by the perception of cytokinin molecules through membrane-associated histidine kinase receptors (AHK2, AHK3, and AHK4/CRE1), which differ in their ligand preferences and tissue-specific expression patterns [14,16,41]. Ligand binding activates a multistep phosphorelay involving *Arabidopsis* histidine phosphotransfer proteins (AHPs) and type-B *Arabidopsis* response regulators (ARRs), which function as transcription factors that rapidly induce primary CK-responsive genes, including type-A ARRs that subsequently provide negative feedback regulation of the pathway [15,41]. The magnitude and timing of transcriptional responses therefore depend not only on cytokinin concentration but also on receptor affinity, ligand stability, metabolism, and downstream signaling dynamics [16,18,41]. Consequently, structurally distinct cytokinins may elicit quantitatively and temporally different transcriptional responses despite activating the same core signaling pathway [18,41].

Unlike the other tested genes in this study, *ARR5* was consistently and strongly (even 15–20-fold at 15 min) upregulated over time under all three treatments, with a significant decline in expression at 24 h. *ARR5* encodes a negative regulator of CK responses; since it participates directly in the CK signaling cascade, it is expected to show high expression levels after exposure to cytokinins [28,29]. It has been shown that *t*Z, BA, and TDZ induce strong upregulation of *ARR5* after 7 days in kohlrabi, although clear downregulation occurred on day 14 with *t*Z [27]. Type-A response regulators similar to *AtARR5* with expression profiles highly responsive to CK application have been found in rice [59] and tomato [60], although direct comparisons should be interpreted with caution because of differences in species, tissue organization, developmental stage, and experimental design. Nevertheless, *ARR5* is also strongly upregulated in *Arabidopsis* by isopentenyladenine (iP), a cytokinin related to *t*Z by its isoprenoid structure [29,61]. It has also been shown that multiple members of the CK signaling cascade, including ARR1 [62] and ARR2 [63], induce direct transcriptional activation of *ARR5*, contributing to a robust, yet complex pattern of transcriptional activation that could explain the different expression profiles obtained in the present study with the various CK treatments.

Our results indicate that after 24 h of CK treatment, the expression levels of *ARR5*, as well as the other analyzed genes, returned to levels similar to those of the control. This pattern may reflect a homeostatic adjustment of CK signaling in response to prolonged hormone exposure, consistent with previous reports [24,64,65,66]. In contrast, other studies [27,29,61] have shown that long-term CK exposure results in persistent changes in gene expression. These differences may indicate that additional regulatory mechanisms become engaged during prolonged hormone treatment [67]; however, the molecular basis of these responses was not investigated in the present study.

The similarity between the expression profiles of *PIN3* and *PIN4* could be explained by their shared role in maintaining cellular auxin homeostasis, as they code for auxin efflux transporters [36]. In our study, these genes were initially downregulated upon exposure to the cytokinins. Furthermore, our results demonstrated that *PIN3* was generally downregulated with *m*T treatment over time, but its expression peaked at 4 h. Růžička et al. [68] showed that a structurally related aromatic cytokinin, BA, positively regulated *PIN3*, but only in the short term, while it was downregulated over the long term. Additionally, Waldie and Leyser [69] found that BA upregulated *PIN3*, *PIN4*, and *PIN7* in the *arr1* genotype of *Arabidopsis*, promoting shoot branching even when a major regulator of CK responses is deficient, suggesting that an alternative mechanism of CK-mediated transcriptional regulation of *PIN3*, *PIN4*, and *PIN7* may exist under these experimental conditions.

Similarly to *m*T, TDZ also induced downregulation of *PIN3* and *PIN4* at most time points, with clear upregulation occurring at 4 and 8 h. In addition, Ćosić et al. [27] reported that TDZ induced the upregulation of both *PIN3* and *PIN4* after 7 days in kohlrabi, but their expression fluctuated during later stages of treatment, displaying several downregulation events. In cotton, the expression of *GhPIN3* responded rather late to the treatment with TDZ, with downregulation occurring at 24 h after treatment [70]. Similarly, our results also showed that TDZ induced a relatively slow response compared to the other treatments, with downregulation occurring after 24 h. Previous studies have shown that cytokinins can induce or repress the expression of *PIN* genes in a spatiotemporal manner, thereby contributing to changes in polar auxin transport in different developmental and tissue-specific contexts [71,72]. Although all three cytokinins in our study mainly led to downregulation of *PIN3* and *PIN4* over time, the intensity and dynamics of the responses varied depending on the cytokinin type.

The other two genes, *STM* and *WUS*, are well-known key master regulators of SAM formation and maintenance, with *STM* being responsible for stimulating cell proliferation and preventing differentiation, while *WUS* maintains stem cell identity in the central zone of the SAM [73]. In our work, several evident *STM* upregulation and downregulation events occurred with all three treatments. The fluctuations between upregulation and downregulation appeared to change from one hour to the next, indicating dynamic expression of this gene. Currently, there is no evidence that cytokinins directly affect *STM* expression [74]. However, it has been shown that transgenic CK-overproducing *Arabidopsis* plants exhibit elevated levels of *STM* mRNA [75]. Additionally, Yanai et al. [76] showed that STM upregulates *AtIPT7*, which encodes an enzyme involved in the biosynthesis of isoprenoid-type cytokinins. Taken together, although direct regulation of *STM* by cytokinins has not been demonstrated, the available evidence suggests that *STM* expression may respond indirectly to CK signaling. The dynamic and variable response of *STM* to *t*Z, *m*T, and TDZ may be associated with indirect regulatory mechanisms, resulting in the alternating upregulation and downregulation of *STM* observed in the present study.

As with *STM*, the *WUS* expression profile exhibited fluctuations over time, with a more or less pronounced downregulation at 2 h following all three treatments, whereas transcript levels increased significantly at 4 and 8 h in response to *m*T and TDZ. *WUS* is known to be positively regulated by cytokinins (particularly iP) through type-B ARRs, which function as upstream transcriptional regulators [77,78,79,80]. Because *WUS* is positioned downstream of the canonical AHK–AHP–ARR signaling pathway, differences in receptor activation kinetics among the applied cytokinins could contribute to the distinct temporal expression profiles observed in this study, although this possibility was not directly investigated. In support of this interpretation, Xie et al. [81] demonstrated binding of ARR1 to the *WUS* promoter after 4 h of BA treatment, which is consistent with the *WUS* expression peak observed with *m*T at 4 h in our study. In contrast, *WUS* transcript levels decreased at two time points following *t*Z treatment. This observation may be related to the findings of Liu et al. [82], who reported that in *Arabidopsis* seedlings treated with iP, ARR1 indirectly represses *WUS* by activating transcription of *IAA17*, a known repressor of auxin-responsive gene expression. Taken together, our results indicate that *WUS* responds differently to the tested cytokinins, with *m*T and TDZ inducing a delayed upregulation, whereas the effects of isoprenoid cytokinins on *WUS* expression appear to be more complex and remain incompletely understood.

The expression levels of *CUC1* and *RGD3* fluctuated greatly over time, with both genes significantly upregulated by *m*T at 4 h and by TDZ at 8 h. By 24 h, their expression levels returned to control levels or even lower with all three treatments. These genes are known to play important roles in SAM formation and shoot growth [34,35]. In kohlrabi, both *CUC1* and *RGD3* were initially upregulated by *t*Z, BA, and TDZ, with TDZ inducing a gradual increase in *CUC1* expression, peaking at day 35 of treatment [27]. This suggests differential regulation of *CUC1* and *RGD3* by isoprenoid, aromatic, and diphenylurea-type cytokinins, although more data from different species and experimental systems are needed to accept this as a generalized rule.

The expression profile of *SHR* was characterized by upregulation events with all treatments, but at different time points. Specifically, *t*Z induced upregulation at 1 h, representing the earliest response, while TDZ was the slowest to initiate *SHR* upregulation, with the peak observed only after 8 h. Unlike adenine-derived cytokinins, TDZ is a phenylurea-type cytokinin with a more complex mode of action. Besides activating CK signaling, TDZ has been reported to influence endogenous CK homeostasis and auxin–cytokinin balance, thereby affecting the timing and magnitude of downstream transcriptional responses [4,15,21,22]. Although these mechanisms were not examined here, they may partly explain the delayed *SHR* response observed following TDZ treatment compared with *m*T and *t*Z. It is not known how cytokinins regulate *SHR* expression; however, previous findings, together with our results, are consistent with the involvement of complex regulatory mechanisms that may include other signaling pathways. For example, Bahafid et al. [37] observed that *SHR* expression is induced by auxin in the SAM via the auxin response factor MONOPTEROS (MP), an important integrator of auxin and CK signals in SAM formation [83]. According to our results, *SHR* expression responds to cytokinin application, but the underlying regulatory mechanism remains unclear and may involve crosstalk between CK and other signaling pathways, with *SHR* mostly downregulated over time, and upregulation events varying in a time-dependent manner depending on the type of applied cytokinin.

Moreover, as *SHR* has been reported to both respond to CK application and influence endogenous cytokinin levels [84,85], the observed expression dynamics may reflect the complex homeostatic regulatory networks controlling CK signaling and metabolism, although the molecular basis of these responses was not investigated in the present study.

The genes *CYCB2;4* and *CDKB2;1* are involved in cell cycle regulation and are expressed during the G2/M transition; thus, they participate in many important developmental processes in plants [38,39,86]. Two distinct expression peaks were observed for both genes: one induced by *m*T at 4 h and another by TDZ at 8 h. In contrast, their responses to *t*Z were noticeably different: *CYCB2;4* showed no significant variation in expression, whereas *CDKB2;1* exhibited two distinct upregulation events, separated by a sharp downregulation at 4 h. As cyclin expression in response to CK requires the cell to be in the active phase of the cell cycle [38], the observed changes in cyclin expression in this study indicate the presence of a sufficient number of mitotically active cells in the shoot tissues of 10-day-old seedlings to detect changes in *CYCB2;4* expression. This also confirms that cell cycle genes are strongly affected by exogenous CK application at the whole-plant level in *Arabidopsis*.

PCA analysis revealed a clear separation of CK treatments at the 4 h time point, indicating distinct transcriptional responses among the applied cytokinins. This observation was further supported by PERMANOVA, which showed that the 4 h time point exhibited the strongest treatment-dependent separation of all analyzed time points, yielding the highest explained variance (R^2^ = 0.678) and F statistic (F = 5.607, *p* = 0.006) [Supplementary Table S2].

Analysis of variable contributions further demonstrated that the first principal component, accounting for 89% of data variability, was driven by the coordinated contribution of all analyzed genes [Supplementary Table S3], suggesting that the observed separation between treatments at 4 h might reflect a general transcriptional response rather than the behavior of a single or few marker genes. In contrast, both the second and third principal components were predominantly influenced by *WUS* and *CUC1*. Together, these findings indicate that, under the experimental conditions used in this study, the 4 h time point represents a duration of cytokinin treatment at which the transcriptional responses of the studied genes to structurally distinct cytokinins are most clearly differentiated.

Although all three cytokinins ultimately affected many of the development-related genes, the pronounced differences in the timing and magnitude of their transcriptional responses suggest that structural variation among cytokinins influences the dynamics of CK signaling rather than only its final transcriptional output. These differences may result from variation in receptor affinity, metabolic stability, transport, and downstream signaling kinetics, all of which have been reported to differ among naturally occurring and synthetic cytokinins. These proposed mechanisms provide a plausible framework for interpreting the distinct temporal expression patterns observed following *t*Z, *m*T, and TDZ treatments and warrant further functional investigation. Previous studies have also shown that different cytokinins can induce distinct physiological and morphogenetic responses. For example, *m*T has been associated with improved shoot quality, whereas TDZ exhibits high morphogenetic potential but may also induce developmental abnormalities due to its high metabolic stability and interference with auxin, gibberellin, and ethylene metabolism and signaling, while *t*Z generally promotes shoot development through the canonical CK signaling pathway [14,15,20,21]. However, whether and how the transcriptional differences observed here translate into distinct developmental outcomes remains to be experimentally validated.

### Study Limitations and Implications for Future Research Directions

The present study has certain limitations that need to be acknowledged.

Firstly, the expression profiles of the ten genes presented in this study are only a small part of the transcriptional landscape affected by the application of different exogenous cytokinins to *Arabidopsis* shoots. The dynamics in the responses of other genes to *m*T, *t*Z, and TDZ might differ from the patterns reported in this work. RNA sequencing experiments need to be employed to dissect both the differences among the effects of different cytokinins within a single time point, and the dynamics with which the application of a particular cytokinin molecule affects the *Arabidopsis* transcriptome over time. Thus, a multidimensional matrix of RNA transcriptomes across different cytokinin treatments, applied concentrations, time points, *Arabidopsis* organs and tissues, or even plant species, is needed in order to comprehensively describe the temporal dynamics of the gene expression responses in various plant tissues to different cytokinin molecules. Our work thus represents only a first effort that illustrates the complexity of the factors that need to be investigated for the comprehensive characterization of the effects of plant hormones on gene expression patterns in plants. It also offers initial insights into the different gene expression profiles induced by different CK treatments, and suggests that at 4 h upon treatment, these differences among different CK treatments might be more pronounced than before or after that, at least in experimental settings similar to the one employed here.

Secondly, a mechanistic explanation for the different effects of the different cytokinins on gene expression is still lacking. The differences in the timing and strength of the gene expression responses induced by different cytokinins are influenced by an array of factors, including differences in the rate of uptake from the nutrient media, affinity for different CK receptors, chemical stability, and metabolic rate (i.e., inactivation or catabolism of different cytokinin molecules) [16,18,87]. Thus, besides the presence of exogenously added cytokinins, endogenous CK pools are also affected by the treatments and participate in CK signaling. Other signaling pathways are also sensitive to these changes, which makes endogenous phytohormone profiling an important next step in mapping the differences between various CK treatments. The observed effects on gene expression profiles are thus the result of a complex homeostatic network in which all of these factors contribute to different extents, and our ability to disentangle these interactions will improve our understanding of the mechanistic effects of exogenous plant hormones on gene expression.

Another limitation of the present study is the use of a single concentration (5 μM) of applied cytokinins, which does not allow for comparison of potential dose-dependent responses between the different cytokinins. The 5 μM concentration was chosen in our work because it has been used in a range of comparative studies on CK-dependent responses on the transcriptomic and proteomic level [24,25,26]. The heterogeneity observed among the expression profiles of all the tested genes in response to the 5 μM concentration of various cytokinins suggests a qualitative divergence among transcriptional outcomes, rather than a simple difference in signal intensity between the different cytokinins. However, because only a single cytokinin concentration was examined here, the observed differences among *t*Z, *m*T, and TDZ cannot be generalized across all concentration ranges, and concentration-dependent responses remain to be investigated.

Lastly, our study demonstrates the limitations of using whole shoots of *Arabidopsis* seedlings as the experimental material. Therefore, the expression profiles reported here should be interpreted as representing average transcriptional responses at the whole-shoot level rather than tissue-specific regulatory events. When using whole *Arabidopsis* shoots for RNA isolation, the contribution of certain tissues, such as mesophyll, to the total RNA is disproportionally high [88]. Consequently, the expression of genes that are relevant to tissue-specific contexts may not be reflected in the obtained results. For instance, the inability to obtain reproducible *CLV3* expression is likely related to its highly restricted expression domain within the central zone of the SAM. Consequently, RNA isolated from whole shoots may have diluted *CLV3* transcripts below reliable detection levels. The example of *CLV3* in this study highlights the importance of selecting the appropriate plant material (shoot, organ, tissue, or single cell) for studying the expression patterns of a particular target gene.

Collectively, these limitations should be considered when interpreting the present findings in order to restrict the conclusions to the analyzed genes, developmental stage, cytokinin concentration, and experimental conditions used in this study.

## 5. Conclusions

Our results indicate that the tested CK structural types are not functionally equivalent with respect to the transcriptional regulation of the analyzed genes under the experimental conditions employed in this study: *m*T induced the strongest transcriptional responses of the tested genes, followed by TDZ and *t*Z. The observed expression patterns are consistent with the involvement of indirect and pleiotropic regulatory processes associated with CK signaling. The observed return to basal expression levels within 24 h further suggests the presence of robust homeostatic regulatory mechanisms. The differential gene expression responses observed here can serve as a basis for further transcriptomic, hormonomic, and other in-depth comparative studies of plant responses to different cytokinin molecules.

## Figures and Tables

**Table 1:** Gene-specific forward (F) and reverse (R) primers used for qRT-PCR.

Gene Name	GenBank™ Accession Number	Primer Sequence	Expected Amplicon Length	TAIR ID
*ARR5*	NM_114679	F: 5′-TCTATGCCTGGGATGACTGGA-3′	175 bp	AT3G48100
		R: 5′-TCACATCAGCTAATTTCACAGGC-3′		
*PIN3*	NM_105762.2	F: 5′-TAAACCAGCGTGATCGGAAG-3′	104 bp	AT1G70940
		R: 5′-ATTGTGCCCTTTGTGTTTGC-3′		
*PIN4*	NM_179592.1	F: 5′-TTGCTTGTGGGAACTCTGTC-3′	129 bp	AT2G01420
		R: 5′-CCTGAACGATGGCTATACGG-3′		
*RGD3*	NM_115288	F: 5′-GACGTGGAGTCATCTAGGCA-3′	162 bp	AT3G54280
		R: 5′-AGCCATCAGCGGCAAGATTA-3′		
*CUC1*	NM_112380	F: 5′-AACGGGACTGAGAACGAACA-3′	189 bp	AT3G15170
		R: 5′-TTGCCGTCAAGGCGATACTC-3′		
*STM*	NM_104916.4	F: 5′-GGGAGCCTCAAGCAAGAGTT-3′	127 bp	AT1G62360
		R: 5′-GCTTTTGTTGCTCCGAAGGG-3′		
*WUS*	NM_127349.4	F: 5′-CAACGTAGGTGGAGGATGGG-3′	137 bp	AT2G17950
		R: 5′-CCAGATAAGCATCGCCACCA-3′		
*CLV3*	NM_128283.2	F: 5′-TGTTGTTCAATTGGCAGATGATG-3′	132 bp	AT2G27250
		R: 5′-CAGGTCCCGAAGGAACAGTC-3′		
*SHR*	NM_119928.3	F: 5′-TCCAAGCTCTCTTCAACCGC-3′	185 bp	AT4G37650
		R: 5′-TGCTTCCAAGATTGCTCCGT-3′		
*CYCB2;4*	NM_106281	F: 5′-GCATCGGCGATCTACACTGC-3′	141 bp	AT1G76310
		R: 5′-CCCTGCCTTGTGATGCAAAC-3′		
*CDKB2;1*	NM_106304	F: 5′-GTACGAGCCAGCGAAACGAA-3′	189 bp	AT1G76540
		R: 5′-GCAGCACACTAGAGATATGCTTGA-3′		
*ACT7*	NM_121018.4	F: 5′-ACAGGAAATGCTTCTAAGTGTGTCT-3′	171 bp	AT5G09810
		R: 5′-ACACAAGACTTCTTGACACAACCA-3′		

## Data Availability

The original contributions presented in this study are included in the article/Supplementary Materials. Further inquiries can be directed to the corresponding authors.

## Supplementary Materials

The following supporting information can be downloaded at: https://www.mdpi.com/article/10.3390/biology15171499/s1, Table S1: Results of the Shapiro–Wilk normality test, Levene’s test for homogeneity of variances, and effect size (partial eta squared, η_p_^2^) for gene expression data at each sampling time point. Normality was assessed separately for each treatment group (control, *trans*-zeatin, *meta*-topolin, and thidiazuron), while homogeneity of variances was evaluated across treatment groups using Levene’s test. Effect sizes were calculated as partial eta squared (η_p_^2^) based on one-way ANOVA performed for each time point. Abbreviations: C, control; *t*Z, *trans*-zeatin; *m*T, *meta*-topolin; TDZ, thidiazuron; Shapiro *p*, *p*-value of the Shapiro–Wilk normality test; Levene test *p*, *p*-value of Levene’s test for homogeneity of variances; η_p_^2^, partial eta squared (effect size). Cells are color-coded using a yellow-to-orange gradient to facilitate visual comparison of values, with darker orange shades representing higher values and lighter yellow shades representing lower values. Table S2: Results of PERMANOVA performed as a complementary multivariate analysis to principal component analysis (PCA) to evaluate differences in overall RT-qPCR gene expression profiles among cytokinin treatments at individual sampling time points. The table reports the coefficient of determination (R^2^), pseudo-F statistic, and permutation-based *p*-value. Abbreviations: R^2^, proportion of variance explained by treatment; F, pseudo-F statistic; *p*, permutation-based *p*-value. Table S3: Contributions (%) of individual genes to the first five dimensions (Dim.1–Dim.5) obtained from the principal component analysis (PCA) of RT-qPCR gene expression data at the 4 h sampling time point. Higher contribution values indicate a greater influence of a given gene on the corresponding PCA dimension. Abbreviations: Dim., principal component (PCA dimension); Contribution (%), percentage contribution of each gene to the corresponding PCA dimension. Cells are color-coded using a blue-to-apricot gradient, where blue shades represent lower contribution values and apricot shades represent higher contribution values.

## Abbreviations

The following abbreviations are used in this manuscript:

*ARR5*	*ARABIDOPSIS RESPONSE REGULATOR5*
*CDKB2;1*	*CYCLIN-DEPENDENT KINASE B2;1*
CK	cytokinin
*CLV3*	*CLAVATA 3*
*CUC1*	*CUP-SHAPED COTYLEDON1*
*CYCB2;4*	*CYCLIN B2;4*
*m*T	*meta*-topolin
*PIN3*	*PIN-FORMED 3*
*PIN4*	*PIN-FORMED 4*
*RGD3*	*ROOT GROWTH-DEFECTIVE 3*
*SHR*	*SHORTROOT*
*STM*	*SHOOTMERISTEMLESS*
TDZ	thidiazuron
*t*Z	*trans*-zeatin
*WUS*	*WUSCHEL*
