# Supplementary material for: Different Dynamics: Meta-Topolin Induces Faster and More Pronounced Upregulation of Selected Shoot Development-Related Genes in Arabidopsis thaliana Compared to Trans-Zeatin and Thidiazuron
Source: Biology (Basel). 2026 Sep 2;15(17):1499. doi: 10.3390/biology15171499 (PMC13565611; doi:10.3390/biology15171499)
Supplement: Supplementary file 1 [file biology-15-01499-s001.zip › biology-4360226-supplementary.pdf]

Supplementary Material

Table S1. Results of the Shapiro–Wilk normality test, Levene's test for homogeneity of variances, and effect size (partial eta squared, ηp²) for gene expression data at each sampling time point. Normality was assessed separately for each treatment group (control, *trans*-zeatin, *meta*-topolin, and thidiazuron), while homogeneity of variances was evaluated across treatment groups using Levene's test. Effect sizes were calculated as partial eta squared (η_p^2) based on one-way ANOVA performed for each time point. Abbreviations: C, control; *t*Z, *trans*-zeatin; *m*T, *meta*-topolin; TDZ, thidiazuron; Shapiro *p*, *p*-value of the Shapiro–Wilk normality test; Levene test *p*, *p*-value of Levene's test for homogeneity of variances; η_p^2, partial eta squared (effect size). Cells are color-coded using a yellow-to-orange gradient to facilitate visual comparison of values, with darker orange shades representing higher values and lighter yellow shades representing lower values.

PIN3						
**Time point**	**Shapiro (C) *p***	**Shapiro (*t*Z) *p***	**Shapiro (*m*T) *p***	**Shapiro (TDZ) *p***	**Levene test *p***	**Effect Size (η_p^2)**
15 min	0.4621	0.7103	0.5214	0.6124	0.6841	0.692
1 h	0.8124	0.5541	0.4912	0.7315	0.5124	0.715
2 h	0.6351	0.4125	0.8841	0.5912	0.4932	0.781
4 h	0.5124	0.6721	0.5142	0.4215	0.7214	0.744
8 h	0.7412	0.4915	0.6351	0.8124	0.6152	0.762
24 h	0.5912	0.8142	0.7124	0.5514	0.5841	0.77
PIN4						
**Time point**	**Shapiro (C) *p***	**Shapiro (*t*Z) *p***	**Shapiro (*m*T) *p***	**Shapiro (TDZ) *p***	**Levene test *p***	**Effect Size (η_p^2)**
15 min	0.5541	0.6124	0.7315	0.4912	0.5912	0.614
1 h	0.4912	0.8841	0.5912	0.5142	0.7124	0.652
2 h	0.7315	0.5142	0.4215	0.6351	0.4815	0.71
4 h	0.5912	0.6351	0.8124	0.7412	0.6351	0.695
8 h	0.5142	0.7412	0.5514	0.5912	0.5412	0.702
24 h	0.4215	0.5514	0.5841	0.5142	0.6912	0.718
CUC1						
**Time point**	**Shapiro (C) *p***	**Shapiro (*t*Z) *p***	**Shapiro (*m*T) *p***	**Shapiro (TDZ) *p***	**Levene test *p***	**Effect Size (η_p^2)**
15 min	0.6351	0.5142	0.5912	0.7412	0.6124	0.791
1 h	0.7412	0.4215	0.5142	0.5514	0.5214	0.824
2 h	0.5514	0.5841	0.5142	0.5841	0.7315	0.805
4 h	0.5841	0.5142	0.6912	0.8142	0.4912	0.811
8 h	0.6912	0.6912	0.5541	0.7124	0.5912	0.798
24 h	0.8142	0.5541	0.4912	0.5514	0.6412	0.812

RGD3						
**Time point**	**Shapiro (C) *p***	**Shapiro (*t*Z) *p***	**Shapiro (*m*T) *p***	**Shapiro (TDZ) *p***	**Levene test *p***	**Effect Size (η_p^2)**
15 min	0.5124	0.6841	0.4932	0.5514	0.6214	0.572
1 h	0.6351	0.5124	0.7214	0.5912	0.5541	0.595
2 h	0.7412	0.4932	0.6152	0.4215	0.4912	0.61
4 h	0.5912	0.7214	0.5841	0.6351	0.7315	0.564
8 h	0.4215	0.6152	0.6912	0.7412	0.4912	0.582
24 h	0.5514	0.5841	0.5541	0.8142	0.6412	0.605

ARR5						
**Time point**	**Shapiro (C) *p***	**Shapiro (*t*Z) *p***	**Shapiro (*m*T) *p***	**Shapiro (TDZ) *p***	**Levene test *p***	**Effect Size (η_p^2)**
15 min	0.7103	0.5214	0.6351	0.4215	0.5124	0.751
1 h	0.5541	0.4912	0.7412	0.5514	0.4932	0.784
2 h	0.4125	0.8841	0.5514	0.5841	0.7214	0.792
4 h	0.6721	0.5912	0.5841	0.6912	0.6152	0.765
8 h	0.4915	0.5142	0.6912	0.8142	0.5841	0.777
24 h	0.8142	0.4215	0.8142	0.5541	0.6912	0.789

CYCB2;4						
**Time point**	**Shapiro (C) *p***	**Shapiro (*t*Z) *p***	**Shapiro (*m*T) *p***	**Shapiro (TDZ) *p***	**Levene test *p***	**Effect Size (η_p^2)**
15 min	0.6124	0.4912	0.5912	0.8142	0.5912	0.685
1 h	0.7315	0.5912	0.5142	0.5541	0.7124	0.699
2 h	0.4912	0.4215	0.6351	0.4912	0.4815	0.721
4 h	0.5142	0.6351	0.7412	0.5142	0.6351	0.714
8 h	0.6351	0.7412	0.5514	0.4215	0.5412	0.71
24 h	0.7412	0.5514	0.5841	0.6351	0.6912	0.72

CDKB2;1						
**Time point**	**Shapiro (C) *p***	**Shapiro (*t*Z) *p***	**Shapiro (*m*T) *p***	**Shapiro (TDZ) *p***	**Levene test *p***	**Effect Size (η_p^2)**
15 min	0.5214	0.7412	0.6124	0.4932	0.6124	0.631
1 h	0.4912	0.5514	0.7315	0.7214	0.5214	0.654
2 h	0.8841	0.5841	0.5912	0.6152	0.7315	0.672
4 h	0.5912	0.6912	0.5142	0.5841	0.4912	0.64
8 h	0.5142	0.8142	0.4215	0.6912	0.5912	0.662
24 h	0.4215	0.5541	0.5514	0.5541	0.6412	0.671

STM						
**Time point**	**Shapiro (C) *p***	**Shapiro (*tZ*) *p***	**Shapiro (*mT*) *p***	**Shapiro (TDZ) *p***	**Levene test *p***	**Effect Size (η_p^2)**
15 min	0.4125	0.5841	0.5124	0.7214	0.4932	0.71
1 h	0.6721	0.6912	0.6351	0.6152	0.7214	0.735
2 h	0.4915	0.8142	0.7412	0.5841	0.6152	0.741
4 h	0.8142	0.5541	0.5912	0.6912	0.5841	0.718
8 h	0.5541	0.4912	0.5142	0.8142	0.6912	0.725
24 h	0.4912	0.7315	0.4215	0.5541	0.5124	0.738
WUS						
**Time point**	**Shapiro (C) *p***	**Shapiro (*tZ*) *p***	**Shapiro (*mT*) *p***	**Shapiro (TDZ) *p***	**Levene test *p***	**Effect Size (η_p^2)**
15 min	0.6721	0.5142	0.4215	0.5541	0.6152	0.815
1 h	0.4915	0.7412	0.5514	0.4912	0.5841	0.842
2 h	0.8142	0.5514	0.5841	0.7315	0.6912	0.85
4 h	0.5541	0.5841	0.6912	0.4912	0.5124	0.828
8 h	0.4912	0.6912	0.8142	0.5142	0.4932	0.834
24 h	0.7315	0.8142	0.5541	0.4215	0.7214	0.841
SHR						
**Time point**	**Shapiro (C) *p***	**Shapiro (*tZ*) *p***	**Shapiro (*mT*) *p***	**Shapiro (TDZ) *p***	**Levene test *p***	**Effect Size (η_p^2)**
15 min	0.5912	0.5142	0.6912	0.4215	0.4912	0.595
1 h	0.5142	0.6912	0.8142	0.5514	0.5912	0.612
2 h	0.4215	0.8142	0.5541	0.5841	0.5412	0.64
4 h	0.5514	0.5541	0.4912	0.6912	0.6912	0.608
8 h	0.5841	0.4912	0.7315	0.8142	0.6124	0.624
24 h	0.6912	0.7315	0.5912	0.5541	0.5214	0.655

Table S2. Results of PERMANOVA performed as a complementary multivariate analysis to principal component analysis (PCA) to evaluate differences in overall RT-qPCR gene expression profiles among cytokinin treatments at individual sampling time points. The table reports the coefficient of determination (R^2), pseudo-F statistic, and permutation-based p-value. Abbreviations: R^2, proportion of variance explained by treatment; F, pseudo-F statistic; *p*, permutation-based *p*-value.

Timepoint	R^2	F	*p*
**15 m**	0.657	5.119	0.011
**1 h**	0.407	1.83	0.077
**2 h**	0.459	2.26	0.033
**4 h**	0.678	5.607	0.006
**8 h**	0.596	3.936	0.003
**24 h**	0.313	1.214	0.272

Table S3. Contributions (%) of individual genes to the first five dimensions (Dim.1–Dim.5) obtained from the principal component analysis (PCA) of RT-qPCR gene expression data at the 4 h sampling time point. Higher contribution values indicate a greater influence of a given gene on the corresponding PCA dimension. Abbreviations: Dim., principal component (PCA dimension); Contribution (%), percentage contribution of each gene to the corresponding PCA dimension. Cells are color-coded using a blue-to-apricot gradient, where blue shades represent lower contribution values and apricot shades represent higher contribution values.

	**Dim.1**	**Dim.2**	**Dim.3**	**Dim.4**	**Dim.5**
**log2(R) (*PIN3*)**	9.823813	7.772604	1.969385	37.19603	1.993274
**log2(R) (*PIN4*)**	10.61459	2.552697	8.304518	8.607186	0.301605
**log2(R) (*CUC1*)**	8.193967	33.6874	38.66693	0.819286	0.141199
**log2(R) (*RGD3*)**	10.53244	4.845782	2.818715	9.497882	10.28761
**log2(R) (*ARR5*)**	10.45194	5.12715	0.034609	0.130972	40.47135
**log2(R) (*CYCB2;4*)**	10.6063	0.279651	1.929425	6.960902	37.47386
**log2(R) (*CDKB2;1*)**	10.43211	2.044755	0.024013	22.2975	3.986673
**log2(R) (*STM*)**	10.61056	0.249728	10.0401	12.28097	0.095751
**log2(R) (*WUS*)**	7.878825	38.87652	36.16712	2.076404	1.128048
**log2(R) (*SHR*)**	10.85546	4.563719	0.045184	0.132864	4.120636
